# A Cross-Sectional Study of Commercial Ewe Management Practices for Different Sheep Breeds across Southern Australia

**DOI:** 10.3390/ani13030388

**Published:** 2023-01-23

**Authors:** Amy L. Bates, Shawn R. McGrath, Maxwell B. Allworth, Susan M. Robertson, Gordon Refshauge

**Affiliations:** 1 School of Agricultural, Environmental and Veterinary Sciences, Charles Sturt University, Wagga Wagga, NSW 2678, Australia; 2 Gulbali Institute, Charles Sturt University, Wagga Wagga, NSW 2678, Australia; 3 New South Wales Department of Primary Industries, Cowra, NSW 2794, Australia

**Keywords:** ewe management, sheep, southern Australia, sheep producers, survey

## Abstract

**Simple Summary:**

Best practice guidelines are available for the management of Maternal and Merino ewes across southern Australia; however, these are lacking for Composite and shedding breeds. Through a telephone interview, the management practices of a unique group of southern Australian sheep producers, and the motivations behind these practices, were explored. A large proportion of respondents reported mating and lambing practices that aligned with best practice guidelines; however, a smaller cohort did not see value in meeting the nutritional and mating length recommendations. Additionally, most producers are also seeking new information frequently and have implemented management changes within the last five years. Further work is required to understand the perceived barriers to best practice adoption, and for management guidelines to be developed for all sheep breeds to ensure sheep enterprises are operating in the most productive and profitable manner.

**Abstract:**

The management of ewes across southern Australia may vary with breed and can change over time and, as such, a greater understanding of producer management practices and the motivations that influence these practices is required. A cross-sectional study was performed by telephone interview with sheep producers managing Composite, Maternal, Merino or shedding ewe breeds mated in either spring, summer, or autumn. The surveyed producers were a unique subset of southern Australian producers. A large proportion of the surveyed producers followed current best practice guidelines for ewe mating and lambing nutritional management; however, some producers did not align with these targets. Further, some producers did not see the value in attaining the current recommendations. Pregnancy scanning was widely practiced, likely an artefact of the recruitment process; however, a few producers did not utilize this information for nutritional management at lambing time. Finally, most producers were active in their search for new information, seeking information regularly from a wide range of sources and reported making management changes within the last five years. Further work is required to understand why some producers are not adopting best practice where possible and to understand current barriers for adoption. Management guidelines for all sheep breeds are required to best manage sheep across southern Australia.

## 1. Introduction

There are multiple breeds of sheep, and management of sheep across Australia depends on many factors. Guidelines have been developed to assist in the management of reproducing Maternal (first-cross breeds) and Merino ewes. It is recommended that Maternal ewes are mated in body condition score (BCS) 3.8–4.2 and at parturition are in BCS 3.0–3.7 (dependent on fetal number) [1]. For Merino ewes, it is generally recommended to mate ewes in BCS 3 and lamb in a similar BCS after regaining the weight lost during early- to mid-pregnancy [2,3]. Sparse research has been conducted on shedding sheep breeds with an industry report exploring Dorper ewe management predicting peak conception when ewes are mated at BCS 3 and above, or 60 kg liveweight and greater [4]. There is no information for Composite ewe management, and thus the management of both shedding and Composite ewe breeds remains relatively unknown in Australia.

Ewe flock management underpins the productivity and profitability of a sheep enterprise and without relevant guidelines and accurate benchmarks for all types of sheep production, flocks may underperform. Economic improvements of up to AUD 0.80/ewe are possible when managing a flock post-scanning, based on fetal number [5]. The month of mating, stocking rate and lamb sale strategy must also be considered together to optimize management [6]. Additionally, there is evidence that breed performance differs when managed according to best practice guidelines, which may influence future progeny performance [7,8]. Economic benefits are associated with a variety of management practices and are therefore important for producers to understand when making management decisions.

Ewe nutritional management is essential to ensure the reproductive success as maiden ewe reproductive rates (fetuses scanned per ewe), lamb survival to marking and lamb marking rates are generally lower and more variable than mature ewes [9]. Separate management of maiden ewes is required for optimal reproductive outcomes of this cohort, improving lifetime productivity [10]. An industry report identified not meeting recommended nutritional targets as a potential barrier to reproductive success in the Australian sheep industry [11]. This is topical as the Australian sheep flock is projected to reach 76 million head in 2022 [12] as the flock rebuilds after an historic population low of approximately 63.5 million head in 2020 [13].

Further, southern Australia produces the largest proportion of sheep, approximately 78% (excluding Western Australia) [14]. Over 53% of producers from different regions across southern Australia reported feed availability as the primary motivator when setting enterprise lambing time [15]. Southern Australia typically experiences a summer and autumn feed gap; however, feed availability varies with seasonal conditions, time of year and geographic region [16] and sheep management practices may change through time [17]. The nutritional management of ewes at mating and throughout pregnancy may have economic consequences for producers and is therefore an important component of sheep production.

The current management guidelines do not provide information for all sheep breeds. The motivations of sheep producers to perform certain management practices, and how these differ, are unknown. Understanding management nuances is essential information for industry to be able to provide sheep producers with effective guidance. As management may change with breed, region and over time, the aims of this cross-sectional study were to report the current management practices of sheep producers across different sheep breeds, and how practices differ between breeds. It was hypothesized that sheep producers managing different ewe breeds would have different management practices.

## 2. Materials and Methods

### 2.1. Study Area, Participants and Survey Design

Sheep producers were recruited through professional networks by the New South Wales (NSW) Department of Primary Industries (DPI) for a broader study [18] in accordance with the Charles Sturt University Human Ethics Committee (protocol number H21037). The producers managing these sheep flocks were the target population and participants/respondents of the cross-sectional study. Inclusion criteria for participation were being over 18 years of age, having one of the specific sheep breeds of interest (Composite, Maternal, Merino and shedding breeds), being located in south-eastern Australia (NSW, Victoria (VIC), South Australia (SA)), ewes being mated in spring, summer or autumn, a minimum flock size of 300 ewes and having mature ewes (i.e., having experienced at least one previous mating event). Participation was voluntary, and responses were recorded anonymously.

The Composite breed is the result of multiple crosses and is primarily a meat producing animal with low wool value but does require shearing. Maternal ewes are first cross ewes with some wool value and are meat-producing. Merino ewes are those with high wool value of various Merino genotypes. Shedding ewes are meat producing hair-based breeds which don’t require shearing and include Dorper, Damara and Australian White.

The survey was conducted by telephone interview between May and July 2021, forming the basis of the cross-sectional study. It was determined by the researchers that a telephone interview would be the most practical method to best capture the geographic spread of producers participating in the survey and to ensure engagement. The structured survey was used to gather information and comprised open (*n* = 26) and closed (*n* = 20) questions (File S1). An iterative process was used to design the survey by the research team (production animal scientists). Three sheep producers piloted the survey to assess the clarity of questions, responses, completion time and relatability of questions to producers, and modifications were made accordingly. It was estimated that the survey would take approximately 30 min to complete. The survey had eight sections:

(i) Demographics and farm structure, focusing on the producer (age (reported in 10-year age brackets from “30 to 39” to “60 to 69” and “over 70”), gender, education and generation), number of ewe and ram breeds, enterprise region and type, flock size, time dedicated to sheep production and pasture information.
(ii) Ewe flock management, such as nutritional assessment of ewes, supplementary feed/feeding (defined throughout as feeding additional feed sources (supplementary feed) to sheep while grazing to increase energy intake to maintain or gain condition) and containment fed/feeding (defined throughout as feeding sheep a feed source (supplementary feed) that constitutes a complete ration in a contained area to maintain or gain condition) practices.
(iii) Mating management questions relating to nutritional mating targets, maiden and mature ewe mating practices, knowledge of short-term feeding prior to mating (referred to henceforth as ‘flushing’), mating season, hormone stimulation and the use of teasers to encourage estrus and ovulation [19]. The use of scoring, either BCS [20,21] or fat score (FS) [22] was also asked.
(iv) Pregnancy scanning management focusing on scanning practices and flock management post-scanning.
(v) Shearing management, including the time of shearing season and rationale behind the selected shearing season.
(vi) Lambing management, such as ewe nutritional targets and ewe condition assessment.
(vii) Weaning management, which addressed factors that influence producer pre-weaning and weaning age management.
(viii) Information sources, such as how, where and how often producers seek information.

### 2.2. Survey Distribution

The potential pool of respondents were producers of Composite, Maternal, Merino and shedding breeds who managed ewe flocks mated during spring (September to November), summer (December to February) or autumn (March to May) in NSW, VIC and SA, comprising approximately 25,199 sheep producers [14].

### 2.3. Data Analyses

Data was collected, checked for entry errors and descriptive analyses performed in Microsoft Excel (2022) [23]. All percentages (%) are reported to the closest integer. Of the closed questions, if several responses reported a specific ‘other’ category, an additional category was created to accommodate this. Likewise, variables with fewer responses were collapsed to create new categories for analytical purposes. Key words in open response questions were used to categories these responses.

## 3. Results

A total of fifty-six (*n* = 56) producers agreed to participate, of which 52 (93%) producers completed the survey. The median call length was 24 min (min) 15 s (s), average time was 27 min 40 s and ranged from 14 min 15 s to 67 min (interquartile range 20 min 0 s to 30 min 45 s).

### 3.1. Demographics

Respondents were predominantly male (94%, 49/52) and mostly located in Central NSW (63%, 33/52). Producers from Northern NSW/Queensland (QLD) (15%, 8/52), Wimmera, Mallee and Murray (10%, 5/52), East Victoria (6%, 3/52) and the SA Peninsula (6%, 3/52) were the remaining respondents. Over 65% (34/52) of producers had tertiary education or university level qualifications in agricultural fields, while 13% (7/52) had completed higher education in a field outside of agriculture and 21% (11/52) had no formal agricultural education. Producers age ranged from 30 to over 70 years and the median age was 49 years. Twenty five percent (13/52) of producers were first or second-generation farmers, 52% (27/52) third or fourth generation and 23% (12/52) fifth generation farmers or greater.

The reproductive ewe flock size ranged from ≤ 1000 ewes to ≥ 6000 ewes and the greatest proportion of producers had between 1001 and 2000 ewes (21%, 11/52). Further breakdown of flock size by breed is available in Table S1. The sheep breeds managed by the respondents were mostly Merino (42%, 22/52) followed by Composite (31%, 16/52), Maternal (19%, 10/52) and shedding (8%, 4/52); however, 21% (11/52) reported having multiple ewe breeds and 40% (21/52) multiple ram breeds. The producers primarily had self-replacing ewe flocks (85%, 44/52), some purchased replacements 13% (7/52), and a single Merino producer (2%) practiced both purchase and self-replacement. Ewe replacement strategy by breed is presented in Table S1.

Most producers identified as ‘predominantly prime lamb’ producers (38%, 20/52), identifying wool as a secondary production output. ‘Prime lamb’ (29%, 15/52), ‘predominantly wool’ (sale of excess animals secondary to wool production, 23%, 12/52) and stud (10%, 5/52) producers made up the remainder of the cohort. The enterprise types are provided in Table 1. Most producers dedicated 81 to 100% of their time to sheep production (Table 1). Overall, 79% (41/52) of producers reported a target lamb sale weight as the determinant of lamb turnoff and of those with a target sale weight 54% (22/41, Table 1) reported that sale weight was dependent on feed availability and seasonal conditions, market prices, age and wool characteristics. Most producers had cropping rotations of fodder and dual-purpose crops (85%, 44/52) with 50% (22/44) of these producers also cropping cereal grains. Of producers with cereal crops, only one (5%) did not graze crop stubbles. Enterprise pasture types (Table 1) are broadly categorized as native pastures (such as *Themeda triandra, Austrodanthonia* spp., *Bothriochloa macra* and *Microlaena* spp.), native shrubs and pastures (such as native pastures and *Maireana* spp. and *Atriplex* spp.), improved pasture (such as *Lolium perenne, Medicago sativa, Lolium multiflorum* and *Trifolium* spp.) and improved native pastures, being improved and native pasture species.

### 3.2. Mating Management

Most producers (92%, 48/52) were aware of the ‘flushing’ practice, where supplementary feed is supplied for a short period of time leading up to mating to improve conception and fetal number [24]. If the term was unfamiliar, it was described as such. Flushing was practiced regularly by 58% (30/52) of producers, and a further 6% (3/52) of producers used the practice dependent on seasonal conditions. Merino producers practiced flushing most frequently (64%), followed by Maternal (60%), Composite (50%) and shedding ewes (50%). Mature ewe flushing length is described in Table 2 on a breed basis and by breed and season in Table S2. Seventy five percent (39/52) of producers across all surveyed breeds thought flushing was a beneficial practice, 19% (10/52) were uncertain if it was beneficial, including Composite, Maternal and Merino producers, and 6% (3/52) thought it was not a beneficial practice, comprising Composite and Maternal producers.

Multiple mating management responses were recorded per respondent. Producers reported the motive for mating time was most often related to the feed requirements of both ewe and lamb (63%, 33/52), followed by market access (i.e., to take advantage of higher prices, 25%, 13/52), environmental conditions (e.g., fly strike, weather extremes and grass seed burden, 21%, 11/52), management practices of other enterprises (15%, 8/52), and fertility (i.e., late summer and autumn, 10%, 5/52). Three Merino producers (6%) reported ‘tradition’, without further explanation, as a reason for managing ewe mating time. Composite (60%, 11/16), Maternal (60%, 6/10) and Merino (68%, 15/22) producers reported feed requirements as the main driver for mating time. Shedding producers were primarily driven by market access (50%, 2/4) and fitting with other management practices (50%, 2/4). Most flocks were mated during summer followed by autumn and then spring. A spring mating was least common among Composite flocks and no surveyed producers managing shedding ewe flocks were mated during summer. The number of ewe flocks joined in each mating season by breed is reported in Table 2.

Maiden ewe age at first mating varied from under 7 months of age, between 7 and 9 months, and between 12 to 18 months, based on liveweight or purchase age (Table 2). Scoring (BCS/FS) was not reported as a mating target for maiden ewes. The age at which maiden ewes were mated differed between breeds. Most Composite ewes and all shedding ewes were mated by 9 months of age compared to approximately 10% of Merino ewes. While there were common maiden mating ages/targets for most ewe breeds, a range of practices were reported by Maternal producers (Table 2). All age/targets were noted by all producers as routine practice, except for one respondent, who reported maiden Merino mating at 12 months rather than 18 months for the first time and planned to continue this practice.

Two respondents (4%, 2/52), one Merino and one Maternal producer, did not mate maiden ewes, and instead purchased mature replacement ewes. Those producers purchasing replacement ewes (8/52) purchased according to age (38%, 3/8), market availability (38%, 3/8) or weight (25%, 2/8). Producers purchasing replacements managed Maternal (75%, 6/8) or Merino (25%, 2/8) ewes. Maiden ewe mating month and length varied by breed, but were predominantly mated during summer (46%, 23/50) for 5 to 7 weeks (80%, 40/50) and are further detailed in Table S1. Maiden ewes were mated separately from mature ewes by 86% of respondents (43/50), with one Maternal producer (2%, 1/50) noting that this was a new practice in the last 12 months and one Merino producer (2%, 1/50) adopted variable practices.

Scoring was utilized by 81% of producers (42/52), being BCS (90%, 38/42) or FS (10%, 4/42). Most producers (71%, 37/52) used scoring at mating as a means of assessing the nutritional status of the ewe. However, of the 10 respondents that did not practice scoring, five respondents (50%) had a score target at mating, three (30%) reported a rising plane or weight-based target and only two (20%) did not have a mating score target. Most producers not scoring were Merino (40%, 4/10), followed by Composite (30%, 3/10), shedding (20%, 2/10) and Maternal (10%, 1/10) producers. Shedding producers were least likely to score (50%, 2/4), followed by Composite (19%, 3/16), Merino (18%, 4/22) and Maternal (10%, 1/10) producers. Conversely, five producers (10%, 5/52) did not have a mating score target, but three (60%, 3/5) practiced BCS up to five times per year. Most producers (87%, 45/52) mated ewes between 5 and 7 weeks in length. The nutritional mating targets and length of mature ewe mating by breed is presented in Table 2.

Mating length for all mature ewe breeds and mating seasons was dominated by a length of 5 to 7 weeks (Table 2). Summer represented the season in which producers were most likely to have a shorter mating period (under 5 weeks), while extended mating periods were reported for all seasons (Table S2). Maiden ewe mating length was predominantly 5 to 7 weeks but differed between breed and season, when compared to mature ewes (Table S3).

Teasing or utilizing the ‘ram effect’ by using vasectomized rams or hormone treated wethers to enhance ovulation, was used by 19% (10/52) of producers. Hormone manipulation to enhance ovulation, especially when mating outside of the ewe’s natural reproductive season, was used by 8% (4/52) of producers. These techniques were utilized most frequently by producers managing Composite ewes (teasing: 50%, 5/10, hormone use: 50%, 2/4). Teaser ram and hormone use by breed is presented in Table S1.

### 3.3. Overall Ewe Flock Management

Seventy five percent (39/52) of producers used both visual ewe assessment (assessment of the physical condition of the ewe visually) and feed available (visual assessment of amount of feed available to the ewe) to assess nutritional requirements and management of their ewe flock. Approximately 81% (42/52) of producers physically scored their ewe flock (either BCS or FS). Of the producers that scored their flock, 74% (31/42) practiced it three or more times per year. Within each breed, at least 50% or more of the respondents scored three or more times per year: 68% of Merino (15/22), 60% of Maternal (6/10) and 50% of Composite (8/16) and shedding (2/4) producers. Visual assessment of feed availability was used independently by 23% (12/52) of producers, mostly Merino (50%, 5/10), followed by Composite (30%, 3/10) and shedding (20%, 2/10) producers. Approximately 19% (10/52) of producers didn’t practice scoring, but the majority (90%, 9/10) of these producers used either visual ewe assessment or feed availability or both to assess the nutritional needs of their flock. Most producers not scoring were Merino (40%, 4/10) followed by Composite (30%, 3/10), shedding (20%, 2/10) and Maternal (10%, 1/10) producers. The proportion of ewes scored within each flock was not recorded. There was a small cohort of respondents (6%, 3/52) that did not believe scoring was a useful practice. Two of these producers were of shedding breeds and one Merino.

Outside of the pre-mating period, most producers practiced supplementary feeding or containment feeding or a combination of both. However, a small portion did not practice supplementary or containment feeding (Table 3). Feed sources used for supplementary and/or containment feeding were by majority cereal grains, primarily barley, followed by conserved forages and legumes (Table S4). Of the 43 producers supplementary and/or containment feeding, 53% used two feed sources and 16% used three feed sources. Reasons for supplementary feeding were to support reproduction (29% 11/38), on a seasonal basis (34%, 13/38) or as needed (39%, 15/38). Containment feeding was practiced for supporting reproduction (7%, 2/29), on a seasonal basis (66%, 19/29), as needed (21%, 6/29), or to conserve ground cover (7%, 2/29). Merino producers were most likely to supplementary feed (86%, 19/22), Composite producers were most likely to containment feed (75%, 12/16) and shedding producers were least likely to practice either (25%, 3/4), respectively. Further, 56% (29/52) of producers had containment feeding infrastructure; however, 14% (4/29) of these producers did not practice containment feeding. Approximately 8% (4/52) of producers indicated plans for containment infrastructure, of which half (2/4) were currently practicing containment feeding using semi-permanent structures.

### 3.4. Scanning and Shearing

Pregnancy scanning was a routine practice for 94% (49/52) of producers, with the majority scanning for fetal number (79%, 41/52), followed by fetal number and fetal age (13%, 7/52) and then pregnancy status (pregnant versus non-pregnant (no detectable fetus)) (8% 4/52). Those not routinely scanning were Merino (67%, 2/3) and Maternal producers (33%, 1/3). The survey did not enquire as to the number of fetuses producers recorded at pregnancy scanning. The main incentive for pregnancy scanning was to manage ewes based on fetal number for differing nutritional requirements (88%, 46/52); however, fertility management (6%, 3/52) and predicting lamb numbers for restocking/sale and feed forecasting (6%, 3/52) were also motives. Management of different ewe cohorts post-scanning was commonplace; however, 4% of respondents (2/52) did not manage their ewe flock based on the pregnancy scanning result. This included one Merino producer who scanned for fetal number and one Composite producer who scanned for pregnancy status.

Most producers (92%, 46/50) allocated better feed/available nutrition to ewes with multiple fetuses, or pregnant ewes (6%, 3/50), while one Merino producer (2%) was unsure how they would manage the different ewe cohorts. Non-pregnant ewes at scanning were retained and culled if no fetus was detected after a subsequent mating event (‘second chance’, 33%, 17/52), sold (35%, 18/52), retained indefinitely (10%, 5/52), or a combination of sale and retainment (23%, 12/52) based on various factors, including season, market, conformation, and age.

Producers with non-shedding ewe breeds (48/52) were predominantly shearing their ewes once annually (85%, 41/48), with a small portion of Merino and Maternal producers shearing every 8–10 months (8%, 4/48) or 6 monthly (6%, 3/48). Respondents reported a variety of reasons for setting their shearing date, which varied by breed and included environment, such as weather extremes, grass seeds and parasites, fitting within management timeframes of other enterprises, reproductive considerations such as fertility, lambing time/survival, and other factors such as tradition, wool quality, and an income injection. The time of year and reason each breed was shorn are presented in Table 3. Regardless of mating season, spring was the most common shearing season followed by summer, and shearing season by breed is further presented in Table S2.

### 3.5. Lambing and Weaning

Most producers (87%, 45/52) reported having a target score (BCS or FS) for their lambing ewes, detailed in Table 3, on a breed basis. However, 27% (12/45) reported this was performed visually, rather than physically scoring the ewe. Nearly half (47%, 21/45) used a physical score and 47% (21/45) reported using both a physical and visual assessment. A single Merino producer monitored ewe weight rather than score at lambing.

Mob size at lambing was considered an important management factor to 83% (43/52) of respondents, 10% (5/52) were unsure and 8% (4/52) did not believe it was an important management practice, which was spread across producers of all ewe breeds. Of respondents who believed lambing mob size was important, 9% (4/43), mostly Merino producers (75%, 3/4), did not believe it had an impact on their own property; however, half of this cohort (50%, 2/4) were unsure what impact mob size had. Despite this, one Merino producer managed single- and multiple-bearing ewes in different sized mobs and one managed maiden mobs at a lower stocking rate compared to mature ewes but did not separate ewes based on fetal number. The perceived impact of mob size on lambing outcome was breed-dependent but was either to improve lamb survival or reduce mismothering (Table 3).

Dividing ewes into different size mobs at lambing based on fetal number was reported by 71% (37/52) of producers, and 18% (9/50) of producers who mated maiden ewes managed them in different sized mobs compared to mature ewes. For mature ewes, mob sizes ranged from 30 to 200 for triplet bearing ewes (14%, 5/37, for producers identifying higher order fetal numbers, rather than ‘multiple’ fetuses), from 50 to 300 for twin or multiple bearing ewes and from 70 up to 1000 head for single bearing ewes, or wherever practical after multiple fetus mobs had been allocated paddocks. Further details are available in Table S5 regarding mature ewe lambing mob size by breed and flock management of maiden ewes at lambing by breed.

The median wean age was 11.9 weeks and mode range was 10 to 16 weeks (Table 3). Multiple responses were provided, but producers primarily weaned lambs based on pasture availability (75%, 39/52), but may also consider the ewes nutritional requirements (50%, 26/52), wean on a set date (38%, 20/52), or lamb liveweight (10%, 5/52). Pasture availability was considered by producers of all breeds; however, weaning on a set date was predominantly performed by Composite (44%, 7/16) and shedding (50%, 2/4) ewe producers. Weaning based on lamb liveweight was performed primarily by Maternal producers (20%, 2/10). 

### 3.6. Information Sources

Respondents sought new information for sheep enterprise management from a variety and combination of sources (multiple responses given, as such, results do not total 100%). This ranged from industry outlets (69%, 36/52, i.e., webinars, industry bodies and management courses) to farm benchmarking groups (40%, 21/52), agricultural professionals (83%, 43/52, i.e., veterinarians, consultants, livestock agents, animal pharmaceutical representatives, government agencies and active researchers), social and print media (27%, 14/52), general internet searches (46%, 24/52) and neighbors (46%, 24/52). Most producers reported making a change to their ewe management in the last five years (85%, 44/52), followed by a change occurring in the last 10 years (8%, 4/52) or more than 10 years ago (8%, 4/52). A range of management changes were reported and can be broadly categorized as reproductive adoptions (44%, 23/52), general flock management (33%, 17/52) and nutritional management (12%, 3/52). Reproductive adoptions included altering the time of mating/lambing, mating ewe lambs, a single mating per year, pregnancy scanning as a management tool, vaccinating against *Campylobacter* spp., hormone manipulation, and retaining ewes based on previous lambing success. General flock management included participating in and implementing best practice guidelines, individual sheep electronic identification, sale of surplus sheep based on conformation, implementing a sound drench and vaccination program, changing breed, weaning lambs earlier and shearing every eight months. Nutritional management included the use of containment feeding, winter-based fodder crops, flushing ewes prior to mating, and mineral supplementation. Most respondents were always seeking information about their sheep enterprise (81%, 42/52), or seeking information on a weekly basis (6%, 3/52), a monthly basis (6%, 3/52), or as needed (8%, 4/52).

## 4. Discussion

The present cross-sectional study elucidated the management practices and the motivation for those practices for 52 sheep producers encompassing four breeds and three mating seasons across southern Australia. The majority of producers were located in the Central NSW region, which coincides with 31% of all southern Australian sheep production (excluding Western Australia) [14]. Merino’s remain the dominant sheep breed across Australia, comprising approximately 68% of the breeding flock [14], and this was reflected in the prominence of Merino producers (42%) in the current study.

The median age (49) and the proportion of female producers (6%) in the participating cohort were both lower than the industry means (59 years and 29%, respectively) for Australia sheep producers [25]. Conversely, the education level of the cohort was higher than previously reported for sheep producers at the last census (65% versus 44%) [25]. Further, the number of producers undertaking ewe pregnancy scanning (94%) was greater compared to previous industry surveys [17,26]. The difference in attributes of the recruited producers compared to Australian sheep producers are possible artefacts of the recruitment process. These differences indicate that the participating cohort was a unique group, biased towards younger producers with higher formal education levels, lower female representation, and a high adoption level of recommended practices.

Scoring sheep to assess the fat and muscle deposition by manual palpation of either the backbone and short rib for BCS [20,21] or the GR site for FS [22] negates the need for weigh scales. Most producers (90%) surveyed used the BCS method, evaluated as a more appropriate scoring alternate to liveweight [27]. The use of BCS and weighing practices align with the Lifetime Ewe Management principles and current industry recommendations for mating management [2,28]. Most Composite and Merino producers aimed for a score of 3.0–3.5 at mating. While there are currently no recommendations for Composite producers in Australia, this aligns with the general recommendation of BCS 3 for Merino ewes [29]. The current recommendations for Maternal ewes are to mate between BCS 3.8–4.2 [1]. However, this was not reported by Maternal producers. It is recommended that shedding ewes have a BCS 3 or liveweight of ≥60 kg at mating [4]; however, no producers reported this target BCS and a single shedding producer reported a weight-based mating target. A few producers did not score ewes and/or did not believe scoring was a useful practice. Given the success and broad uptake of the Lifetime Ewe Management course [29], understanding the mating targets across a broad range of sheep breeds is important to understand what sheep producers currently are or are not practicing.

The economic benefit of managing the nutritional requirements of ewes based on pregnancy scanning results (fetal number) has been demonstrated [5]. The number of producers routinely pregnancy scanning within Australia is unknown; however, it is likely around 45% [17,30,31]. The higher pregnancy scanning rates of the current study is likely an artefact of the recruitment process. A range of management practices post-scanning have also been reported [30] and align with those recorded in the current survey. Further, region may influence pregnancy scanning practice [17], but data was limited for conclusion from the present study. A small proportion of non-shedding producers did not manage ewe flocks based on pregnancy scanning results, which may lead to reduced enterprise productivity and profitability.

The most profitable sheep production system, determined via bio-economic modelling, was one with a diverse income portfolio that decreased risk through varied production outputs (sheepmeat and wool) [32]. This is consistent with the large portion of producers that reported managing multiple ewe and ram breeds or practicing mixed enterprise farming. Producers also practiced variable weaning and lamb sale strategies in agreeance with further modelling work [6], dependent on available feed, seasonal conditions, and market prices, which would further spread risk.

Further, bio-economic modelling has also shown that the management of ewe liveweight profiles, especially at mating, was most profitable [3]. It was optimal for Merino ewes to lamb in the same condition as at mating, achieved through supplementary feeding when necessary [3], and for Maternal ewes to lose up to 1.3 BCS between mating and lambing [1]. An industry report for Dorper ewes recommended following Merino guidelines if spring lambing, and otherwise to consider both the cost of feeding and liveweight loss on lamb performance [4]. Producers across the current study generally aimed to lamb ewes in a similar condition to mating. This could economically disadvantage non-Merino flocks if supplementary feeding to attain these higher targets was required. The method to achieve the target lambing condition, however, was not reported in the current survey.

Ewe mating management practices were driven by a combination of factors and aligned with a previous survey [15]. Producers of spring-mated flocks had the least representation in the current study. This may reflect the recruitment process, be due to natural breeding season of sheep [33], or the typical feed gap associated with the late summer–early autumn lambing season [15,16]. However, out-of-season reproductive deficits can be overcome with adequate nutrition [34], and/or reproductive hormone manipulation [35]. Use of the ‘ram effect’, which results in the stimulation of reproductive hormones through exposure to male pheromones [19], may also be an effective tool, but may occur without the knowledge of the producer when rams are initially introduced to the ewe flock at the commencement of mating. Hormone manipulation and the ‘ram effect’ were adopted most commonly in Composite breeds in this study.

The success of mating maiden ewes can be highly variable; however, can optimize lifetime productivity and profitability if managed correctly [36,37]. Estrus may be less regular and shorter in maiden ewes when compared to mature ewes [38], and as such, mating maiden and mature ewes separately is recommended [2]. In the current survey, the majority of Composite and shedding ewe producers mated maiden ewes at 7 to 9 months of age. This may reflect the success of this genotype to reach the critical liveweight or condition required for successful mating [10]. Further, the successful mating of ewe lambs in low- or nil- wool-value enterprises allows ewe lambs to contribute income sooner. Liveweight, but not BCS, was reported as a target for mating maiden ewes but may be due to the framing of the question *(“… age … first join maiden ewes*”). Conversely, few Merino producers engaged in mating ewe lambs, but it may be possible to improve Merino ewe lamb reproduction through the management of liveweight and with higher ram percentages [39,40]. Further, the mixed mating practices reported by Maternal producers were unexpected, but this cohort predominantly purchased replacement ewes and the mixed practices are likely related to market access and/or availability. Region may influence these practices, but collected data was too sparse to draw conclusions.

The provision of additional feed may be required to meet sheep nutrient and energy demands and mitigate gaps in feed supply [41]. Most producers reported using supplementary and/or containment feeding during both the pre-mating period and outside of this time, but this practice varied by breed. The proportion of producers reporting plans to build or update containment feeding infrastructure may indicate forward planning for enterprise resilience. The practice of flushing ewes prior to mating can improve estrus response and ovulation rate dependent on the type of supplement and the length of time fed [42,43,44]. A range of flushing lengths were reported, which is consistent with a previous survey [45], and varied by breed. A proportion of producers, predominantly autumn and summer mated Merino producers, were uncertain or did not believe flushing was a beneficial practice. This experience may be the result of the type of supplement used, length of time fed [42], or that peak reproductive potential was already realized.

Larger lambing mobs [46] and higher lambing stocking rates may reduce lamb survival across breeds, with the effect greatest in twin-born lambs [47]. Further, producers may be underestimating the impact of lamb mortality on production [48]. In the current study, most producers reported divided lambing mobs by fetal number. The perceived impact of lambing mob size varied by breed and was occasionally unknown. The survey did not explore whether producers separated mature and maiden ewes at lambing, which may be associated with reduced maiden lamb survival [49]; however, the majority of producers practiced separate maiden and mature ewe mating. The survey also did not explore the minimum mob size producers perceived as appropriate. A range of mob sizes were reported, and while this may impact lamb survival, further information around paddock structure and size is required.

Producers reported a range of motives for shearing time and included environmental conditions, fitting with other enterprise management practices, reproduction, and availability of shearers, likely related to both breed and region. Regarding reproduction, the impact of shearing during pregnancy is varied. Shearing at day 70 or 120 of gestation reportedly improved lamb vigor (standing and suckling behaviors) [50]. However, shearing at day 90 or 130 resulted in lambs with slower reactions to behavioral tests, but it may be beneficial in mitigating the effect of a perinatal cold challenge [51].

Information sources identified by respondents aligned with a previous survey [17]. Most producers sought information all the time and had made changes to their sheep enterprise management within the last five years, being predominantly reproductive management changes, which is a novel finding. However, these results may be linked to the unique group of producers included in the survey.

The current cross-sectional study provides an insight into current ewe management practices of southern Australian sheep producers. However, there were limitations. Producer demographics differed from the average southern Australian sheep producer. As such, causal relationships can only be hypothesized. Extrapolating findings from the current cross-sectional study for broad application must be approached with caution as the results are not considered generalizable. This is likely an artefact of the recruitment process and voluntary nature of the survey, reducing representation of southern Australian sheep producers [25]. It is recommended that future surveys explore the management of non-pregnant ewes and those not pregnancy scanning or scoring to gain further insight into the motives behind producer practice. Region and season may also influence management practices and motives and may reveal further nuances; however, data was too sparse for conclusions. The participating producers are likely to be those with a connection to researchers within NSW DPI, or to other producers or consultants with this connection, and may reflect the higher use of best practice guidelines.

## 5. Conclusions

Most producers surveyed followed best practice guidelines for the management of ewes at mating and lambing. Producers of different ewe breeds reported different management practices at mating, lambing and nutritional management. Understanding the differences in ewe management at the breed level is important, especially as management practices may alter with time. The findings of this cross-sectional study highlight the need for breed specific guidelines to ensure sheep enterprises are both productive and profitable and provide a valuable insight into the way sheep producers are accessing and using available information. Identifying the barriers to best practice adoption is required to ensure producers can make the best decision for their enterprise.

## Figures and Tables

**Table 1:** Demographic characteristics of southern Australian sheep producers with response reported as a percentage (%, to the closest integer) of respondents (*n* = 52) with count (*n*) in parentheses.

Category	Response % (*n*)
Enterprise type	
Sheep	56 (29)
Sheep and cattle	8 (4)
Sheep and cropping	8 (4)
Cattle	6 (3)
Cropping	19 (10)
Other ^A^	4 (2)
Dedicated time for sheep	
up to 40%	14 (7)
41% to 60%	25 (13)
61% to 80%	15 (8)
81% to 100%	46 (24)
Lamb sale weight	
Up to 18 kg	10 (5)
18.1 to 20 kg	21 (11)
20.1 to 24 kg	31 (16)
Over 24.1 kg	17 (9)
No target weight	21 (11)
Pasture type	
Improved	12 (6)
Native	17 (9)
Improved native	67 (35)
Native shrubs and pasture	4 (2)

^A^ Sheep are not a large component of overall enterprise.

**Table 2:** Mating management practices of southern Australian sheep producers by breed. Responses are recorded as counts (*n*) and overall percentage (%, to the closest integer) of respondents. A dash (‘-’) indicates no data.

Mating Management Practice	Breed				Overall %
	Composite (*n*)	Maternal (*n*)	Merino (*n*)	Shedding (*n*)	
Flush Length ^A^ (*n* = 30)					
Up to 2 weeks	4	1	4	-	30%
Up to 4 weeks	3	-	4	2	30%
Up to 6 weeks	-	2	2	-	13%
Up to 8 weeks	-	3	1	-	13%
12 weeks or more	-	-	2	-	7%
No routine	1	-	1	-	7%
Mature ewe mate month (*n* = 52)					
Spring	1	2	7	2	23%
Summer	8	6	8		42%
Autumn	7	2	7	2	35%
Maiden ewe age mating target (*n* = 50)					
Under 7 months of age	-	-	-	1	2%
7 to 9 months of age	15	2	2	3	44%
12 to 18 months of age	1	1	19	-	42%
Weight	-	2	-	-	4%
Age ^B^	-	4	-	-	8%
Mature ewe mating condition target (*n* = 52)					
Below score 3	4	3	2	2	21%
Score 3 to 3.5	7	2	13	-	42%
Score 3.5 to 4	1	-	2	1	8%
Weight based	-	2	1	1	8%
Rising plane (no BCS ^C^ target)	2	1	3	-	12%
No target	2	2	1	-	10%
Mature ewe mating length (*n* = 52)					
Under 5 weeks	1	1	-	-	4%
5 to 7 weeks	15	7	19	4	87%
7 to 10 weeks	-	-	3	-	6%
Over 10 weeks	-	2	-	-	4%

^A^ Flush length refers to the practice of short-term supplementary feeding pre-mating. ^B^ Age of maiden ewes available when purchasing replacements. ^C^ BCS = body condition score.

**Table 3:** Management practices of southern Australian sheep producers by breed. Responses are recorded as counts (*n*) and overall percentage (%, to the closest integer) of respondents. A dash (‘-’) indicates no data.

Management Practice	Breed				Overall %
	Composite (*n*)	Maternal (*n*)	Merino (*n*)	Shedding (*n*)	
Feeding practices (*n* = 52)					
Supplementary fed	2	6	6	-	27%
Containment fed	2	1	2	-	10%
Supplementary and containment fed	10	1	12	1	46%
Neither supplementary nor containment fed	2	2	2	3	17%
Shearing season (*n* = 48)					
Summer	5	3	7	-	31%
Autumn	4	1	3	-	17%
Spring	7	5	5	-	35%
Winter	-	1	6	-	15%
No set shearing time	-	-	1	-	2%
Reasons for shearing season ^A^ (*n* = 48)					
Environmental ^B^	6	4	9	-	40%
Management ^C^	12	8	8	-	58%
Reproduction ^D^	10	3	13	-	54%
Shearer availability ^E^	3	2	9	-	29%
Other ^F^	3	2	5	-	21%
Target condition at lambing (*n* = 52)					
Below Score 3	3	1	1	1	12%
Score 3 to 3.5	8	6	14	1	56%
Score 3.5 to 4	3	2	4	1	19%
No target score	2	1	2	1	12%
Weight	-	-	1	-	2%
Perceived impact of mob size (*n* = 42)					
Lamb survival	7	1	12	1	44%
Mismothering	6	7	5	1	40%
Uncertain	2	1	4	1	17%
Lamb weaning age (*n* = 52)					
8 weeks (no variation)	-	1	2	1	8%
8 to 12 weeks	5	2	4	1	23%
10 to 16 weeks	8	5	14	1	54%
Over 16 weeks	3	1	-	-	8%
Dependent on seasonal conditions	-	1	2	1	8%

^A^ Multiple responses recorded per producer. ^B^ Includes weather extremes, grass seeds, and parasites (flies, lice). ^C^ Includes wider enterprise management requirements and fits shearing within this. ^D^ Includes mating and/or lambing time, and/or lamb survival. ^E^ Availability of shearers each season determined when ewes were shorn. ^F^ Other factors included an injection of income between other enterprise sales, tradition and for wool quality.

## Data Availability

The data that support this cross-sectional study cannot be publicly shared due to ethical or privacy reasons and may be shared upon reasonable request to the corresponding author if appropriate.

## Supplementary Materials

The following supporting information can be downloaded at: https://www.mdpi.com/article/10.3390/ani13030388/s1, File S1—Producer survey and File S2—Supplementary material; Table S1: Total reproductive ewe flock numbers (no.) by breed and mating management practices of southern Australian sheep producers. Responses are recorded as counts (*n*) and overall percentage (%, to the closest integer) of respondents. A dash (‘-’) indicates no data; Table S2: Management practices of southern Australian sheep by breed and mating season. Responses are recorded as counts (*n*) and overall percentage (%, to the closest integer) of respondents. A dash (‘-’) indicates no data; Table S3: Maiden ewe mating length of southern Australian sheep producers by breed and mating season. Responses are recorded as counts (*n*) and overall percentage (%, to the closest integer) of respondents. A dash (‘-’) indicates no data; Table S4: Feed sources used by southern Australian sheep producers, including supplementary feed sources when supplementary and/or containment feeding. Responses are reported as a percentage (%, to the closest integer) with count (*n*) in parentheses. Multiple feed sources were reported by some producers (total response % exceeds 100); Table S5: Lambing management practices of southern Australian sheep producers by breed. Responses are recorded as counts (*n*) and overall percentage (%, to the closest integer) of respondents. A dash (‘-’) indicates no data.
