# Supplementary material for: A Cross-Sectional Study of Commercial Ewe Management Practices for Different Sheep Breeds across Southern Australia
Source: Animals (Basel). 2023 Jan 23;13(3):388. doi: 10.3390/ani13030388 (PMC9913841; doi:10.3390/ani13030388)
Supplement: Supplementary file 1 [file animals-13-00388-s001.zip › animals-2162613-supplementary.pdf]

Table S1. Total reproductive ewe flock numbers (no.) by breed and mating management practices of southern Australian sheep producers. Responses are recorded as counts (*n*) and overall percentage (%, to the closest integer) of respondents. A dash ('-') indicates no data.

Category	Breed				Overall %
	Composite (%)	Maternal (%)	Merino (%)	Shedding (%)	
Total sheep no. (*n* = 52)					
Up to 1000	2	3	5	-	19%
1001 to 2000	2	2	5	2	21%
2001 to 3000	3	2	3	2	19%
3001 to 4000	2	1	5	-	15%
4001 to 6000	2	2	4	-	15%
6001 and above	5	-	-	-	10%
Ewe replacement strategy (*n* = 52)					
Self-replace	16	4	20	4	85%
Purchase replacement ewes	-	6	1	-	13%
Both self-replace and purchase	-	-	1	-	2%
Maiden ewe mating month (*n* = 50)					
Spring	-	-	7	1	16%
Summer	7	7	8	1	46%
Autumn	9	2	6	2	38%
Maiden ewe mating length (*n* = 52)					
Under 5 weeks	1	1	-	-	4%
5 to 7 weeks	12	6	18	4	80%
7 to 10 weeks	3	1	3	-	14%
Over 10 weeks	-	1	-	-	2%
Teaser ram use (*n* = 10)	5	3	2	-	100%
Hormone use (*n* = 4)	2	1	1	-	100%

Table S2. Management practices of southern Australian sheep by breed and mating season. Responses are recorded as counts (n) and overall percentage (%, to the closest integer) of respondents. A dash ('-') indicates no data.

Management practice	Mating season			Overall %
	Spring (n)	Summer (n)	Autumn (n)	
**Flushing length (mature ewe, n = 30)**				
Composite (n = 8)				
Up to 2 weeks	-	2	2	50%
Up to 4 weeks	-	3	-	38%
No routine	-	1	-	13%
Maternal (n = 6)				
Up to 2 weeks	-	1	-	17%
Up to 6 weeks	-	2	-	33%
Up to 8 weeks	1	1	1	50%
Merino (n = 14)				
Up to 2 weeks	2	2	-	29%
Up to 4 weeks	-	1	3	29%
Up to 6 weeks	-	1	1	14%
Up to 8 weeks	1	-	-	7%
12 weeks or more	1	-	1	14%
No routine	1	-		7%
Shedding (n = 2)				
Up to 4 weeks	1	-	1	100%
**Mature ewe mating length (n = 52)**				
Composite (n = 16)				
Under 5 weeks	-	1	-	6%
5 to 7 weeks	1	7	7	94%
Maternal (n = 10)				
Under 5 weeks	-	1	-	10%
5 to 7 weeks	2	4	1	70%
Over 10 weeks	-	1	1	20%
Merino (n = 22)				
5 to 7 weeks	5	7	7	86%
7 to 10 weeks	2	1	-	14%
Shedding (n = 4)				
5 to 7 weeks	2	-	2	100%
**Mature ewe shearing month (n = 48)**				
Summer	4	4	7	31%
Autumn	1	5	2	17%
Winter	1	2	4	15%
Spring	3	11	3	35%
No routine	1	-	-	2%

Table S3. Maiden ewe mating length of southern Australian sheep producers by breed and mating season. Responses are recorded as counts (*n*) and overall percentage (%, to the closest integer) of respondents. A dash ('-') indicates no data.

**Mating length (*n* = 50)**	**Mating season**			**Overall %**
	**Spring (*n*)**	**Summer (*n*)**	**Autumn (*n*)**	
Composite (*n* = 16)				
Under 5 weeks	-	1	-	6%
5 to 7 weeks	-	3	9	75%
7 to 10 weeks	-	3	-	19%
Maternal (*n* = 9)				
Under 5 weeks	-	1	-	11%
5 to 7 weeks	-	4	2	67%
7 to 10 weeks	-	1	-	11%
Over 10 weeks	-	1	-	11%
Merino (*n* = 21)				
5 to 7 weeks	5	7	6	86%
7 to 10 weeks	2	1	-	14%
Shedding (*n* = 2)				
5 to 7 weeks	2	-	2	100%

Table S4. Feed sources used by southern Australian sheep producers when supplementary and/or containment feeding. Responses are reported as a percentage (%, to the closest integer) with count (*n*) in parentheses. Multiple feed sources were reported by some producers (total Response % exceeds 100).

**Feed source**	**Response % (*n*)**
Cereal grains	
Available grain	9 (4)
Barley	60 (26)
Corn	5 (2)
Corn based pellets	7 (3)
Oats	23 (10)
Triticale	2 (1)
Wheat	5 (2)
Wheat based pellets	2 (1)
Legumes	
Available beans	2 (1)
Faba beans (*Vicia faba L.*)	5 (2)
Lucerne	5 (2)
Lupins	19 (8)
Conserved forage	
Hay	14 (6)
Silage	7 (3)
Available supplement [A]	2 (1)

[A] Available supplement is defined as using feed sources that are available at the time of supplementary feeding, having no specific preference.

Table S5. Lambing management practices of southern Australian sheep producers by breed. Responses are recorded as counts (*n*) and overall percentage (%, to the closest integer) of respondents. A dash ('-') indicates no data.

Category	Breed				Overall %
	Composite (*n*)	Maternal (*n*)	Merino (*n*)	Shedding (*n*)	
**Mature ewes**					
Single flock size at lambing (*n* = 37)					
Up to 100	1	-	2	-	8%
101 to 200	1	1	4	-	16%
201 to 400	5	6	6	1	49%
500 to over 1000	-	1	2	-	8%
DSE basis [A]	1	-	1	-	5%
Single flock	4	-	1	-	14%
Multiple/twin flock size at lambing (*n* = 37)					
Up to 100	3	5	9	1	49%
101 to 200	4	1	3	-	22%
201 to 400	3	1	3	-	19%
DSE basis	2	1	1	-	11%
Triple flock size at lambing (*n*= 5)					
Up to 50	2	-	-	-	40%
51 to 100	1	-	-	-	20%
101 to 200	2	-	-	-	40%
**Maiden ewes**					
Flock management at lambing (*n* = 9)					
Up to 200	1	1	1	-	33%
201 and above	1	-	-	-	11%
Separate singles and twins	2	-	1	-	33%
Separate triplets	1	-	-	-	11%
Lower DSE	1	-	-	-	11%

[A] DSE = Dry Sheep Equivalent, a 50 kg dry sheep
